# Supplementary material for: Content-rich biological network constructed by mining PubMed abstracts
Source: BMC Bioinformatics. 2004 Oct 8;5:147. doi: 10.1186/1471-2105-5-147 (PMC528731; doi:10.1186/1471-2105-5-147)
Supplement: Additional File 2 — The original results of the above study (non-essential files are deleted to keep the file size under the limit set by BMC bioinformatics). [file 1471-2105-5-147-S2.bz2 › chilibotAdditionalFile2/dip05/302ID8647433E1293/html/U2AF1_U2AF2.html]

 


 **U2AF1** and **U2AF2** 
  
Found 123 abstracts in PubMed, retrieved 05.  
 

 What does Google say? 
 PDF only 
| .edu only 

---

**Interactive relationship** (e.g. stimulation, inhibition, etc)

**Neutral relationship**- The splicing factor U2AF  [ **U2AF2** ]   [ **U2AF1** ]  65, U2 small nuclear ribonucleoprotein particle snRNP auxillary factor of 65 kDa, binds to pyrimidine rich sequences at 3 splice sites to recruit U2 snRNP to pre mRNAs.  Ref: 12558503 Biochem J, 2003

**Non-interactive relationship** (e.g. studied together, co-existance, homology, etc.)

- The arginine and serine rich domain of U2AF  [ **U2AF2** ]   [ **U2AF1** ]  65 is critical for U1 recruitment, and we discuss the role of its RNA RNA annealing activity in this novel function of U2AF  [ **U2AF2** ]   [ **U2AF1** ]  65.  Ref: 12558503 Biochem J, 2003
- It inserts a conserved tryptophan into a hydrophobic pocket between helices A and B in a way that strikingly resembles part of the molecular interface in the U2AF  [ **U2AF2** ]   [ **U2AF1** ]  heterodimer.  Ref: 12718882 Mol Cell, 2003
- Structural Basis for the Molecular Recognition between Human Splicing Factors U2AF  [ **U2AF2** ]   [ **U2AF1** ]  65 and SF1 mBBP.  Ref: 12718882 Mol Cell, 2003
- The structure of the C terminal RRM RRM3 of human U2AF  [ **U2AF2** ]   [ **U2AF1** ]  65 complexed to an N terminal peptide of SF1 reveals an extended negatively charged helix A and an additional helix C.  Ref: 12718882 Mol Cell, 2003
- It also appears that some RNA processing factors are required for viability e.g., U2AF  [ **U2AF2** ]   [ **U2AF1** ] , PUF60, SRp54, SAP49, PRP8, U1 70K.  Ref: 12810918 RNA< MedlineTA>RNA, 2003
- The structural basis for recognition between human splicing factors U2AF  [ **U2AF2** ]   [ **U2AF1** ]  65 and SF1 mBBP has recently been elucidated, setting the scene for understanding a key step in pre mRNA splicing and revealing a new conserved function for RRM proteins.  Ref: 12737811 Structure Camb , 2003
- Over expression of either TcSR or the human SR protein associated splicing factor splicing factor 2 ASF SF2 in wild type Schizosaccharomyces pombe, provoked an elongated phenotype similar to that of fission yeast over expressing the SR containing splicing factor Prp2, a U2AF  [ **U2AF2** ]   [ **U2AF1** ]  65 orthologue.  Ref: 12615334 Mol Biochem Parasitol, 2003
- We report that U2AF  [ **U2AF2** ]   [ **U2AF1** ]  65 can also promote the recruitment of U1 snRNP to weak 5 splice sites that are followed by uridine rich sequences.  Ref: 12558503 Biochem J, 2003
- The essential splicing factors SF1 and U2AF  [ **U2AF2** ]   [ **U2AF1** ]  play an important role in the recognition of the pre mRNA 3 splice site during early spliceosome assembly.  Ref: 12718882 Mol Cell, 2003
